# Normal Proportions of the Horse

**Published:** 1843-11

**Authors:** 


*Normal Proportions of the Horse.*—In the French school of veterinary art, the height of the horse, measured from the poll of the head to the ground, is estimated at three head's length; from the top of the withers to the ground, at two and a half ditto; the distance from the point of the shoulder to the point of the quarter at two and a half heads' length; the height from the summit of the croup to the ground at

2 38.100.

From the summit of the withers to the point of immersion of the neck in the throat 66.100, and the same from the withers in a horizontal line, to a level with the summit of the croup, extending the same horizontal line, to a level with the point of the quarter; and from the point of one shoulder to that of the other in a straight line.

From the immersion of the neck in the withers to the mane, half a head's length; and to the point of the shoulder 82.100 of a head's length; and the same from the point of the quarter to that of the stifle; from one haunch to the other in a direct line; from the summit of the croup to the stifle; from the stifle to the hock; and from the hock to the ground.

The greatest breadth of the belly, in a straight line, equal to one head's length; the depth of the body from the lowest part of the back to its greatest dip, the same. Depth from the summit of the withers to the lowest dip of the chest, one head 22.100; from the withers to the stifle, one head 64.100; and the same from the summit of the croup to the elbow. Eclipse had not these proportions; he measured 66 inches (16½ hands) in height, standing an inch higher behind than before, and this great height was still exceeded by the length of his body, that being 69 iuches. He was remarkable, however, for the smallness of his head, that being only 22 inches in length, or one-third instead of two-fifths of his height. "Eclipse was never esteemed handsome, yet he was swift, and the mechanism of his frame was perfect," says Saint-Bel, who accordingly set up his proportions for a model in the English veterinary school, Eclipse having been unquestionably a race-horse of the first distinction both for speed and bottom; and who won more renown on the turf than any horse either before or since his day.—*London Lancet*, from *Veterinarian*, August.

